# Rapid Electron Acceleration in Low‐Density Regions of Saturn's Radiation Belt by Whistler Mode Chorus Waves

**DOI:** 10.1029/2019GL083071

**Published:** 2019-07-08

**Authors:** E. E. Woodfield, S. A. Glauert, J. D. Menietti, T. F. Averkamp, R. B. Horne, Y. Y. Shprits

**Affiliations:** ^1^ British Antarctic Survey Cambridge UK; ^2^ Department of Physics and Astronomy University of Iowa Iowa City IA USA; ^3^ Helmholtz Centre Potsdam, GFZ German Research Centre for Geosciences Potsdam Germany; ^4^ Institute for Physics and Astronomy Universität Potsdam Potsdam Germany; ^5^ Department of Earth, Planetary, and Space Sciences University of California Los Angeles CA USA

**Keywords:** Saturn, radiation belt, electron, wave‐particle interaction, whistler mode chorus waves

## Abstract

Electron acceleration at Saturn due to whistler mode chorus waves has previously been assumed to be ineffective; new data closer to the planet show it can be very rapid (factor of 10^4^ flux increase at 1 MeV in 10 days compared to factor of 2). A full survey of chorus waves at Saturn is combined with an improved plasma density model to show that where the plasma frequency falls below the gyrofrequency additional strong resonances are observed favoring electron acceleration. This results in strong chorus acceleration between approximately 2.5 R_S_ and 5.5 R_S_ outside which adiabatic transport may dominate. Strong pitch angle dependence results in butterfly pitch angle distributions that flatten over a few days at 100s keV, tens of days at MeV energies which may explain observations of butterfly distributions of MeV electrons near L=3. Including cross terms in the simulations increases the tendency toward butterfly distributions.

## Key Points

- Very low plasma densities found at Saturn significantly enhance the electron acceleration due to whistler mode chorus waves
- Extra diffusion where the plasma frequency/gyrofrequency <1 leads to rapid formation of butterfly pitch angle distributions
- Chorus acceleration of electrons at Saturn is an important mechanism for radiation belt dynamics between 2.5 R_S_ and 5.5 R_S_

## 1 Introduction

The electron radiation belts at Saturn until recently were thought to be the result of the production of high‐energy electrons from Cosmic Ray Albedo Neutron Decay and radial diffusion of lower‐energy seed population electrons (Kollmann et al., 2011; Kollmann et al., 2018; Roussos et al., 2018). Recently, however, Woodfield et al. (2018) showed that wave‐particle interactions with Z‐mode waves have a very important role in accelerating electrons inside the orbit of Enceladus. Whistler mode chorus waves, which are a key source of electron acceleration at the Earth (Horne et al., 2005) and Jupiter (Horne et al., 2008; Woodfield et al., 2014), were dismissed as having negligible effect on the electrons at Saturn due to the combination of plasma and magnetic field conditions (Lorenzato et al., 2012; Shprits et al., 2012). These two studies investigated the effect of chorus waves at radial distances greater than 5.5 R_S_.

The long duration of the Cassini mission has increased our understanding of the plasma conditions and wave characteristics at Saturn compared to the earlier assumptions used in Shprits et al. (2012) and Lorenzato et al. (2012), and we have revisited the question of the effect of chorus waves on the energetic electron population. In this paper we investigate wave particle interactions from 2.5 R_S_ to 7.5 R_S_ and show that chorus waves are very effective at accelerating electrons inside of 5.5 R_S_. We also show that the acceleration is heavily dependent on pitch angle and that this could lead to butterfly pitch angle distributions (PADs).

## 2 Plasma Density and Chorus Wave Intensity

The ratio of the plasma frequency to the gyrofrequency, f_pe_/f_ce_, is very important for electron acceleration through wave‐particle interactions, with a value of f_pe_/f_ce_<4 resulting in good electron acceleration (Horne et al., 2003). The region of interest here is between 2.5 R_S_ and 7.5 R_S_, and it is reasonable to assume the magnetic field to be dipolar this close to Saturn (Carbary et al., 2010) where the effects of the magnetodisc are small. A centered dipole is used with an equatorial surface magnetic field strength, B_0_, of 2.1951×10^−5^ T.

We use the plasma density model from Persoon et al. (2015) which uses the scale height formulation from Persoon et al. (2006) with updated parameters. In comparison to the Thomsen et al. (2010) model used by Shprits et al. (2012) the equatorial electron density in Persoon et al. (2015) is very similar but the scale height is much smaller so very low densities are reached at comparatively low latitudes.

The rapid decrease in density with latitude agrees with the wave observations. In their survey of chorus waves between 4.5 R_S_ and 7.5 R_S_ Menietti et al. (2014) set a latitudinal cutoff value for chorus observations at 25° to avoid regions dominated by the lower frequency whistler mode hiss waves. Assuming chorus is observed at frequencies above 0.05 f_ce_ and hiss at frequencies below 0.05 f_ce_ then chorus will cease to be present leaving only the lower frequencies of the hiss whistler mode above 24° at 4.5 R_S_ using the Persoon et al. (2015) density model. The equivalent cutoff using the Thomsen et al. (2010) model would be 50° at the same radial distance. Therefore, a smaller‐scale height model fits much better with the wave data that are observed.

The chorus wave power survey in Menietti et al. (2014) covered radial distances from 4.5 R_S_ to 7.5 R_S_; in this paper we have extended the lower range of the survey to 2.5 R_S_ using similar methodology to Menietti et al. (2014; see supporting information). We use the wave power in the magnetic field given by equation (1)

(1) $$P(\lambda ,f)=P(\lambda )\exp(-{\left(f-f_{m}\right)}^{2}/f_{w}^{2})$$

where P(λ) is the wave power variation with latitude; and f, f_m_, and f_w_ are the wave frequency, frequency of maximum wave power, and frequency width with all frequencies relative to the equatorial gyrofrequency. The values used in this paper are in Table 1; the values from 4.5 R_S_ to 7.5 R_S_ are taken from Figure 6 of Menietti et al. (2014). The Gaussian form of chorus wave power with latitude does not fit well inside of 4.5 R_S_; hence, a linear variation of wave power with latitude is used.

**Table 1:** Parameters Used for the Chorus Waves in the Simulations

Radial distance	Wave intensity (using λ in °)	f_m_	f_w_	f_uc_	f_lc_
(R_S_)	(nT^2^)	(f_ce_)	(f_ce_)	(f_ce_)	(f_ce_)
2.5<R<4.5	−2.099×10^−8^ λ + 2.946×10^−6^	0.31	0.14	0.50	0.10
4.5 ≤ R<5.5	1.01163×10^−5^exp(−((λ−11.489)/17.177)^2^)	0.30	0.19	0.50	0.05
5.5 ≤ R<6.5	7.09282×10^−5^exp(−((λ−8.541)/5.226)^2^)	0.30	0.19	0.50	0.05
6.5 ≤ R ≤ 7.5	3.21251×10^−5^exp(−((λ−7.138)/5.577)^2^)	0.30	0.19	0.50	0.05

**Figure 1:**
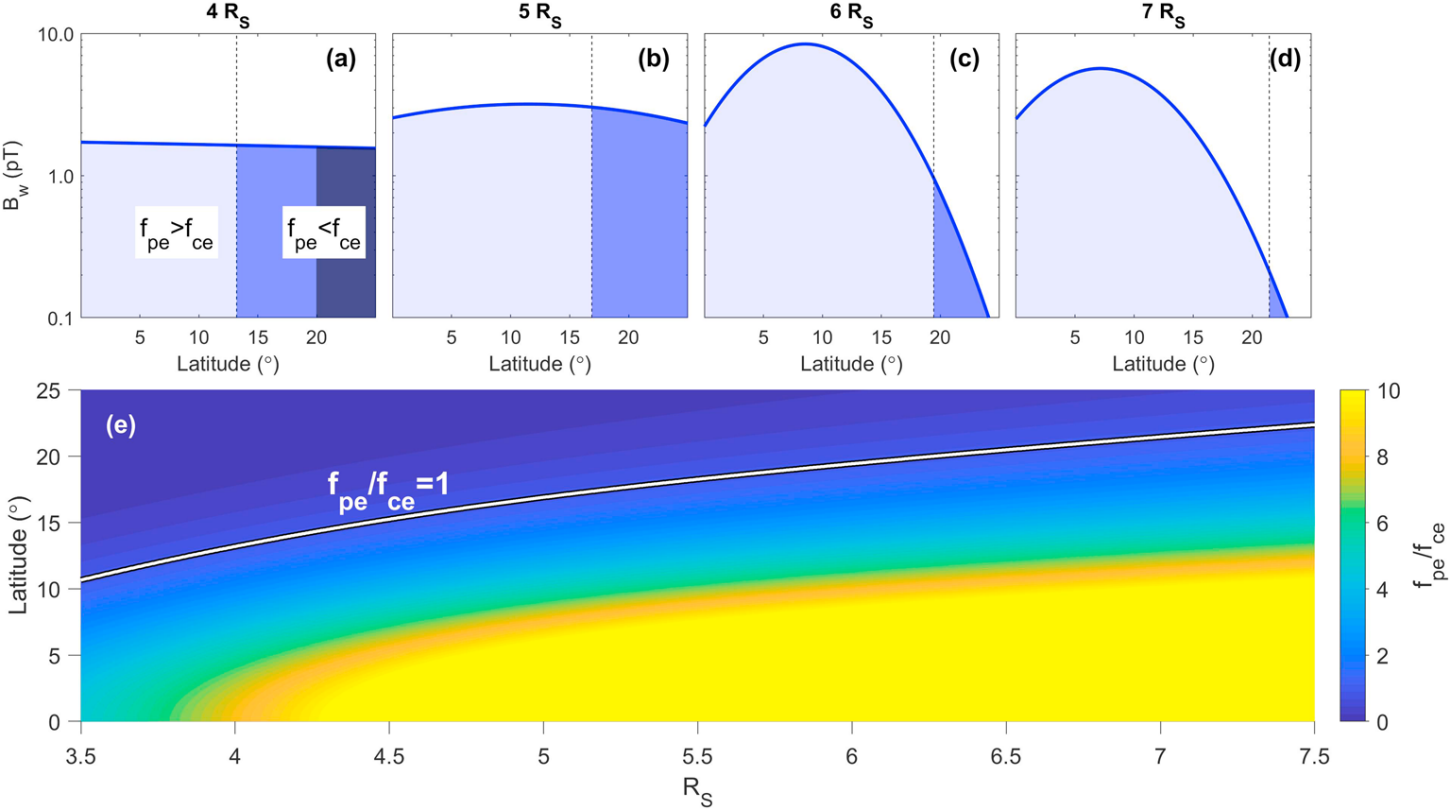
Distribution of wave power and the ratio of plasma frequency, f_pe_, to gyrofrequency, f_ce_, with latitude. (a–d) Wave power with latitude at four different Saturn radii with regions of f_pe_>f_ce_ shown in pale blue, regions of f_pe_<f_ce_ in darker blue. (a) The darkest blue region is where f_pe_<f_ce_/10 where the whistler mode is below the frequency cutoff we use for chorus (see Table 1). (e) The ratio f_pe_/f_ce_ versus latitude and radial distance.

Figure 1 shows how f_pe_/f_ce_ and wave amplitude vary with radial distance. A change in the character of the chorus power with latitude is observed around 5.5 R_S_; outside of this location the power is much more confined in latitude. The effect of changes to the chorus power often observed in interchange events at Saturn (Paranicas et al., 2016) are restricted to the region 5.5 R_S_ to 6.5 R_S_(Menietti et al., 2014).

## 3 Modelling the Effect of Wave‐Particle Interactions on the Electrons

To isolate the local effect of the waves on the electrons in terms of their energy and pitch angle, we use quasi‐linear methods to solve the modified Fokker‐Planck equation without radial diffusion (equation (2); Glauert et al., 2014; Woodfield et al., 2014).

(2) $$\begin{array}{ccc} \frac{\partial f}{\partial t} & =\frac{1}{g(\alpha )}{\left.\frac{\partial }{\partial \alpha }\right|}_{E,L}g(\alpha )\left(D_{\alpha \alpha }{\left.\frac{\partial f}{\partial \alpha }\right|}_{E,L}+D_{\alpha E}{\left.\frac{\partial f}{\partial E}\right|}_{\alpha ,L}\right) & \\ & \;+\frac{1}{A(E)}{\left.\frac{\partial }{\partial E}\right|}_{\alpha ,L}A(E)\left(D_{EE}{\left.\frac{\partial f}{\partial E}\right|}_{\alpha ,L}+D_{E\alpha }{\left.\frac{\partial f}{\partial \alpha }\right|}_{E,L}\right)-\frac{f}{\tau } \end{array}$$

where

(3) $$g(\alpha )=\sin2\alpha \left(1.3802-0.3198(\sin\alpha +{\left(\sin\alpha \right)}^{\frac{1}{2}})\right)$$

(4) $$A(E)=(E+E_{0}){\left(E(E+2E_{\circ })\right)}^{\frac{1}{2}}$$

where *f* is the phase space density; *t* is time; *α* is the equatorial pitch angle; and *D*_*αα*_, *D*_*EE*_, and *D*_*αE*_ are the drift‐ and bounce‐averaged pitch angle, energy, and cross diffusion coefficients, respectively. *E* is the energy, *E*_0_ is the electron rest energy, and *τ* is the atmospheric loss timescale (dependent on *α* and *E*). The cross‐diffusion coefficients are identical with *D*_*αE*_=*D*_*Eα*_.

The British Antarctic Survey radiation belt model (BAS RBM) uses implicit methods to solve equation (2). The grid resolution is 90×90 points (*α*, *E*), the energy grid uses equal spacing in the natural log of the energy, and we use a time step of 500 s.

The cross terms are included in the calculations using alternating direction implicit methods, specifically the Hunsdorfer‐Verwer scheme (in ‘t Hout & Welfert, 2007) with nine‐point discretization.

We use isotropic electron flux data from Tang and Summers (2012) to set the flux at *α*=90° at the minimum energy boundary ( $E_{\min}=40$ keV), and the flux at the maximum energy boundary ( $E_{\max}=50$ MeV) is set at 1×10^−7^ cm^−2^·s^−1^·sr^−1^·keV^−1^ (10 times lower than the minimum observed in the data). We use the condition *∂f*/*∂α*=0 at both the minimum and maximum *α* boundaries. The initial condition is set to be a straight line (on a log‐log plot) from the flux at $E_{\min}$ to the flux at $E_{\max}$ with a sin(α) PAD similar to published pancake distributions (Carbary et al., 2011; Clark et al., 2014). This represents an initial seed population, from particle injections for example. A straight line is used rather than a step function as the initial acceleration from a step function is extremely fast due to the large gradient in energy (Woodfield et al., 2014). We use a loss cone angle which depends on the radial distance at atmospheric height (assumed to be 1,000 km, likely an overestimate due to Saturn's oblateness, and planet radius of 60,330 km). Inside the loss cone *τ* is one quarter of the bounce time and infinite elsewhere.

### 3.1 Diffusion Coefficients

**Figure 2:**
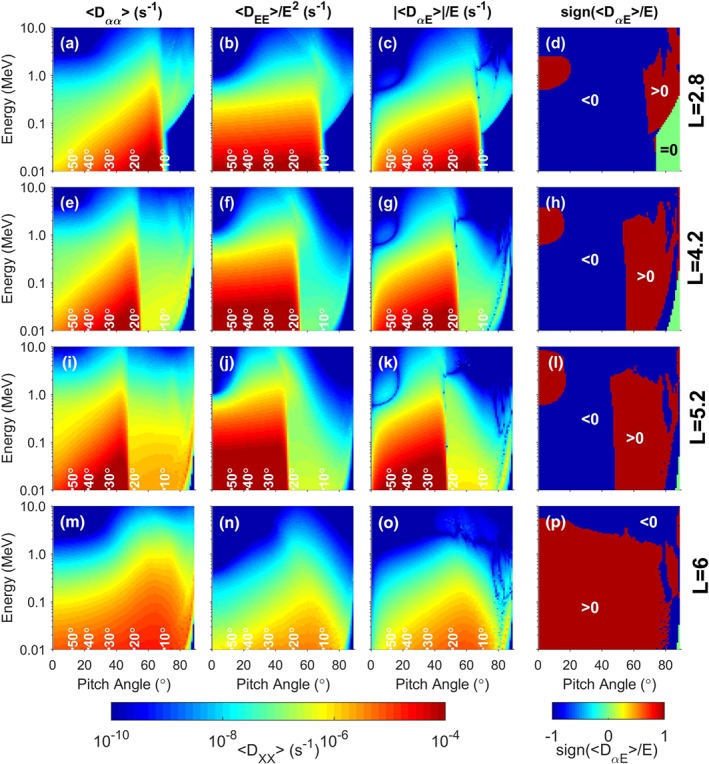
Bounce and drift‐averaged diffusion coefficients at L=2.8, 4.2, 5.2, and 6.0 (top to bottom rows, respectively). (a, e, i, m) <D_αα_>, (b, f, j, n) <D_EE_>/E ^2^, (c, g, k, o) |<D_αE_>/E|, and (d, h, l, p) the sign of <D_αE_>/E.

The diffusion coefficients shown in Figure 2 are calculated using the PADIE code (Glauert & Horne, 2005) which solves the resonance condition for cold plasma dispersion in a magnetic field. We use the wave properties from Table 1 combined with the plasma and magnetic field models from section 2. We assume the waves are field aligned with a peak wave normal angle of 0° and width of 30° with lower and upper cutoffs at 0° and 60°. We calculate the bounce and drift average of the diffusion coefficients assuming the waves are uniform in local time and present from 0° to 25° latitude. We use the McIlwain *L* shell parameter and choose *L* shell values to be over 20 moon radii away from the moons to clearly avoid any extra effects on the chorus wave power that may be introduced by the moons (Santolik et al., 2011; Shprits et al., 2018). Note that the cross diffusion term can be positive or negative, whereas the other diffusion terms are always positive.

A significant change occurs between *L*=5.2 and *L*=6.0 with lower *L* shells giving rise to much larger diffusion coefficients, in particular, a marked increase for *α*<∼50° at *L*=5.2. The increase is largest in the energy diffusion and will potentially lead to butterfly‐shaped PADs. This extra diffusion comes from the region where *f*_*pe*_/*f*_*ce*_<1 (highlighted in Figure 1). From the equator up to where *f*_*pe*_/*f*_*ce*_=1 there is weak diffusion across a wide range of *α* at all the *L* shells shown (similar to Shprits et al., 2012, for *L*=6). This is visible at the highest *α* at all *L* shells shown in Figure 2 but becomes the dominant feature at *L*=6.0 where the additional diffusion from low densities is very weak.

The primary reason for the change between *L*=5.2 and *L*=6.0 at Saturn is the relative proportion of wave magnitude greater than ∼0.1 pT in the *f*_*pe*_/*f*_*ce*_<1 region. Most of the diffusion for the *f*_*pe*_/*f*_*ce*_<1 region is where the cyclotron resonance, *n* (Glauert & Horne, 2005), is 0, although there are smaller contributions from up to *n*=±15 and beyond. We have limited our calculations to *n*=±15 since the contributions at higher *n* are increasingly small.

Where *f*_*pe*_/*f*_*ce*_<1 whistler mode waves can approach the resonance cone where the waves change character from electromagnetic to electrostatic. PADIE uses the magnetic wave power as the wave power input, and we have therefore included an additional factor to remove electrostatic wave power which would lead to erroneous diffusion coefficients (see supporting information).

To include the contribution to pitch angle scattering from collisions with the atmosphere (important close to the loss cone) we calculate a pitch angle diffusion coefficient using the method of Abel and Thorne (1998) and profiles of atmospheric constituents from Moore et al. (2009; see supporting information).

There are several other mechanisms that can cause losses and scattering in Saturn's electron radiation belts: scattering due to planetary rings and neutral molecules, absorption by moons, and potentially other wave‐particle interactions. Our aim in this paper is to isolate the effect of the chorus waves as far as possible so that their contribution to the radiation belt dynamics can be understood. This is an advantageous approach since chorus can then be added to radiation belt models in a modular way.

## 4 Simulation Results

**Figure 3:**
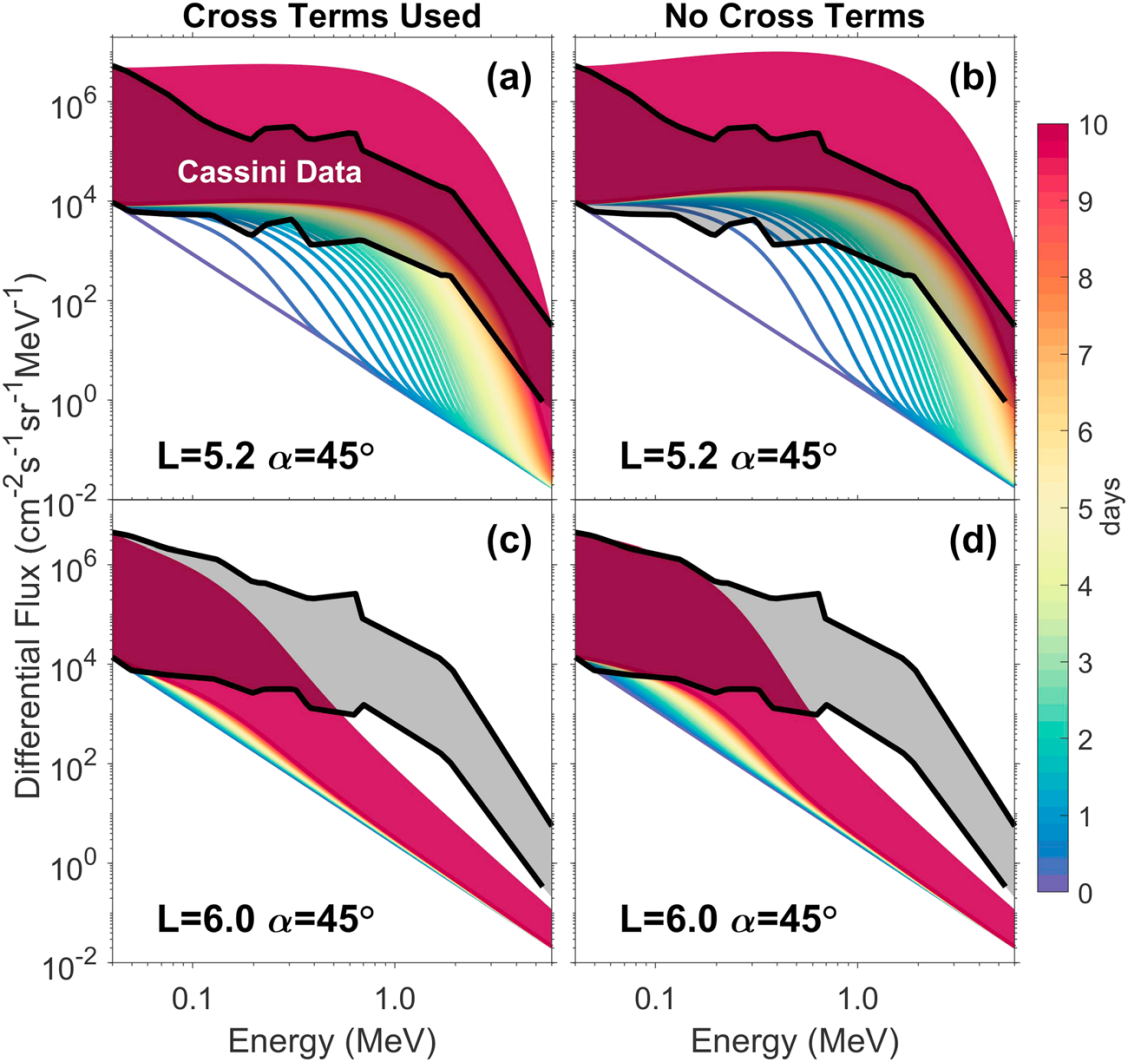
Simulations of pitch angle and energy diffusion with and without the cross terms, (a and b) at L=5.2 and (c and d) at L=6.0. Differential electron flux evolves from the initial condition (darker blue line) through yellow to finally red lines (lines are separated by 0.2 days). Broad red band shows the range of possible fluxes after 10 days due to uncertainties in starting conditions. Cassini data extremes from Tang and Summers (2012) are shown as black lines with gray in between.

Figure 3 shows the results of BAS RBM simulations at *L*=5.2 and *L*=6.0 over 10 days with and without cross terms included (approximately steady state at *L*=5.2). Cross terms are sometimes neglected to simplify the numerical schemes required to solve equation (2). It is typically assumed that the effect of cross terms is most apparent at lower *α* and that they reduce the rate of electron energy increase by around an order of magnitude at *α*=90° (Shprits et al., 2012; Tao et al., 2009).

To cover the range of possible values of flux at $E_{\min}$, we perform two simulations: one using the smallest value of flux and one the largest from Figure 2 of Tang and Summers (2012). Assuming the flux at $E_{\min}$ varies over time between these two extreme values, the range of possible end points of the simulation after 10 days is shown as the broad red band in each panel of Figure 3. Increasing (decreasing) the flux at $E_{\min}$ produces a very similar shaped energy spectrum over time but shifts the whole spectrum up (down) in magnitude (Woodfield et al., 2014).

Figures 3a and 3c show the dramatic difference the extra diffusion present at lower *L* shells makes to the energy diffusion. Figures 3b and 3d show the same comparison but with the cross terms neglected. As expected the cross terms result in slower electron acceleration at both *L*=5.2 and *L*=6.0.

To produce an approximately steady state energy spectrum comparable to that at *L*=5.2 after 10 days but at *L*=6.0 would take approximately 50 times as long. These results show that acceleration due to chorus is sufficient to reach observed levels but tends to result in a flatter energy spectrum below 1 MeV. This may indicate the need for losses in less idealistic simulations.

## 5 Pitch Angle Distribution Changes

**Figure 4:**
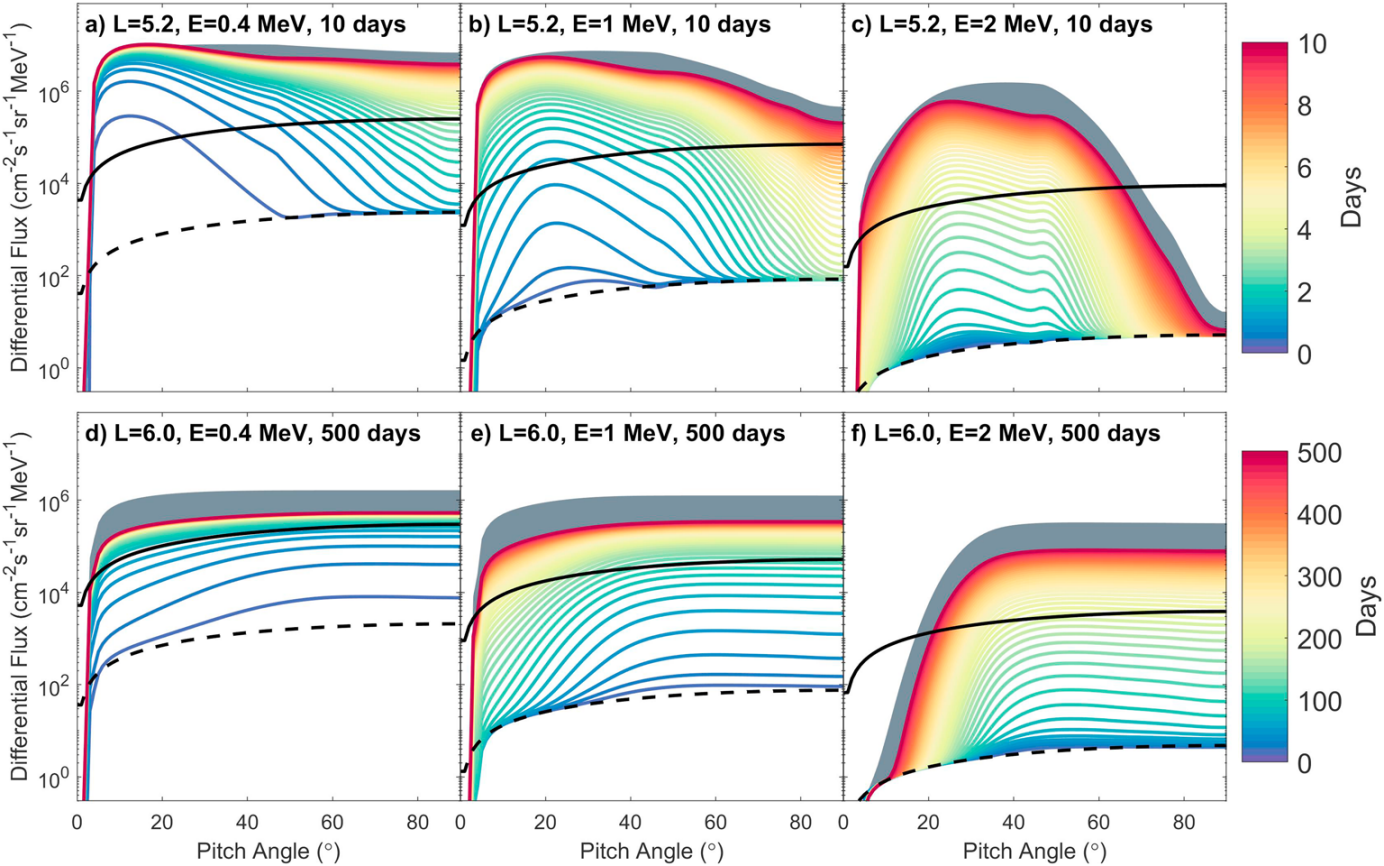
Pitch angle distributions from L=5.2 and 6.0. Tang and Summers (2012) data used with imposed sin(α) dependence, no loss cone, and ∂ f/∂ α=0 at 0° and 90° (solid black line) and initial simulation condition derived from it (dashed black line). Cross terms simulation is in color, gray behind shows the final few days of same simulation without cross terms. L=6.0 run (d–f) is for 50 times longer than L=5.2 run (a–c).

At *L*=5.2 with strong and pitch angle‐dependent diffusion from the low‐density region the acceleration at lower *α* is very rapid. Figures 4a–4c show the PADs at *L*=5.2 for energies of 0.4, 1.0, and 2.0 MeV. The color lines show the PADs evolving over time when cross terms are included. The gray shaded region shows the effect of neglecting the cross terms.

The simulation at *L*=5.2 rapidly forms butterfly PADs with fluxes exceeding the observed data demonstrating the importance of including chorus waves in radiation belt simulations but also of including losses to reduce the fluxes produced. The line separation in Figures 4a–4c is 0.2 days. These asymmetric distributions smooth out to fill in the higher *α* with a rate dependent on energy; at 2 MeV there is still a butterfly distribution after 10 days. For 2‐MeV electrons to reach a distribution similar to Figure 4a for 0.4‐MeV electrons would take ∼100 days.

The effect of the cross terms on the PADs at *L*=5.2 is to increase the asymmetry further by reducing the acceleration most at mid–pitch angles (particularly evident in Figure 4c). This is different to the cross‐term effect on the distributions at *L*=6.0 which is very similar across all *α* (Figures 4d–4f). This demonstrates the importance of including the cross terms in all calculations as they depend on the individual characteristics of the wave and the underlying plasma and magnetic conditions.

## 6 Summary and Discussion

The low‐density conditions that occur at Saturn lead to background plasma conditions that are very different to those usually encountered where chorus waves are observed at the Earth (Meredith et al., 2003). Where *f*_*pe*_/*f*_*ce*_<1 a separate and distinct pattern of diffusion coefficients is found. At Saturn, due to the scale height of the Enceladus torus, this low‐density region begins at typically 10° to 20° latitude. The latitudinal dependence of the density leads to a strong pitch angle dependence in the diffusion coefficients.

The presence of highly pitch angle‐dependent diffusion results in rapidly formed butterfly PADs that flatten out over time, over days at lower energies (0.4 MeV) and tens of days at megaelectron volt levels. Clark et al. (2014) show Cassini observations of PADs at ∼0.4 MeV at *L*=5.13. The average PAD observed in Clark et al. (2014) is very close to a pancake distribution as is also typical at energies lower than this (Carbary et al., 2011). Additionally, Clark et al. (2014) show that the individual PADs at 5 *R*_S_ are predominantly pancake shaped. We interpret the lack of observed butterfly PADs at lower energies as a combination of chorus waves being present most of the time in addition to loss processes that are also present in the region. At higher energies, butterfly PADs have been observed near *L*=3 (Paranicas et al., 2010; see supporting information), and we propose this is due to chorus waves.

The effective lifetime of the electrons defined by Kollmann et al. (2011), $f/(\frac{\partial f}{\partial t})$, is calculated here for the pitch angle and energy diffusion of a 1 MeV electron using equation (2) assuming no sources and no losses, resulting in effective lifetimes of approximately 10^6^ s at *L*=5.2 and 10^8^ s at *L*=6.0. These compare to the effective lifetime of radial diffusion from the average observed data of ∼10^8^ s (found from Figure 5, curve 2 and equation 17 of Kollmann et al., 2011, to estimate effective lifetime at 200 keV, then extrapolate to 1 MeV using Figure 8 of the same paper). The shorter effective lifetime due to chorus waves at *L*=5.2 shows that chorus acceleration is rapid compared to radial diffusion and the presence of other waves need to be taken into account to fully reproduce the steady state.

Losses could be due to collisions with the neutral torus and ring particles (which are slow, continuous losses; Woodfield et al., 2018; Lorenzato et al., 2012) or as yet unquantified wave‐particle interactions, particularly scattering due to whistler mode hiss (Menietti et al., 2014) and ion cyclotron waves (Leisner et al., 2011; Meeks et al., 2016). Recent results outside of 4 *R*_S_(Kollmann et al., 2018) show that electron energy spectra are time variable on top of a trend that follows the effect of radial diffusion; some of this variation may be due to chorus wave acceleration and subsequent losses.

We find that the typically assumed form of influence of cross terms, that is, strongest effect at low pitch angles, is not true for the chorus waves in the low‐density region where acceleration is reduced most at midrange pitch angles. The overall effect of the cross terms is approximately an order of magnitude reduction of flux, similar to previous studies (Shprits et al., 2012; Tao et al., 2009).

Our results show that the influence of chorus waves is likely to be strong inside of ∼*L*=5.5 but minimal beyond (in agreement with previous studies outside 5.5 *R*_S_; Lorenzato et al., 2012; Shprits et al., 2012). However, a more detailed consideration is required of the often enhanced wave activity observed in interchange regions (Tao et al., 2011) and near some moons (Santolik et al., 2011; Shprits et al., 2018). Chorus waves are a significant part of the dynamics of the electron radiation belts at Saturn which need to be included in future 3‐D studies involving multiple source and loss processes.

## Supporting information



Supporting Information S1
