# Supplementary material for: Rapid Electron Acceleration in Low‐Density Regions of Saturn's Radiation Belt by Whistler Mode Chorus Waves
Source: Geophys Res Lett. 2019 Jul 8;46(13):7191–8. doi: 10.1029/2019GL083071 (PMC6772095; doi:10.1029/2019GL083071)
Supplement: Supplementary file 1 — Supporting Information S1 [file GRL-46-7191-s001.pdf]

Supplementary Information

Rapid electron acceleration in low density regions of Saturn's radiation belt by whistler mode chorus waves

E. E. Woodfield, S. A. Glauert, J.D. Menietti, T.F. Averkamp, R. B. Horne, and Y.Y. Shprits

Survey of Saturn Chorus Data between L=2.5 and L=4.5

We give here a summary of the survey data for whistler mode chorus waves in the region inside of 4.5 R_S which is not included in the Survey of Menietti *et al*. [2014]. This data has not been published elsewhere and covers the time range 15th January 2005 to 26th April 2017. The data has been processed in the same way as in Menietti *et al*. [2014].

Supplementary Figure 1 shows the variation of wave power with latitude over all local times and the L-shell range 2.5< L <4.5. This data has been used to produce a linear weighted least squares fit to the variation with latitude

$$P_\lambda = m_\lambda \lambda + c_\lambda$$

Where P_λ is the wave power averaged over latitude, m_λ is the gradient and c_λ is the intercept (see Supplementary Figure 1).

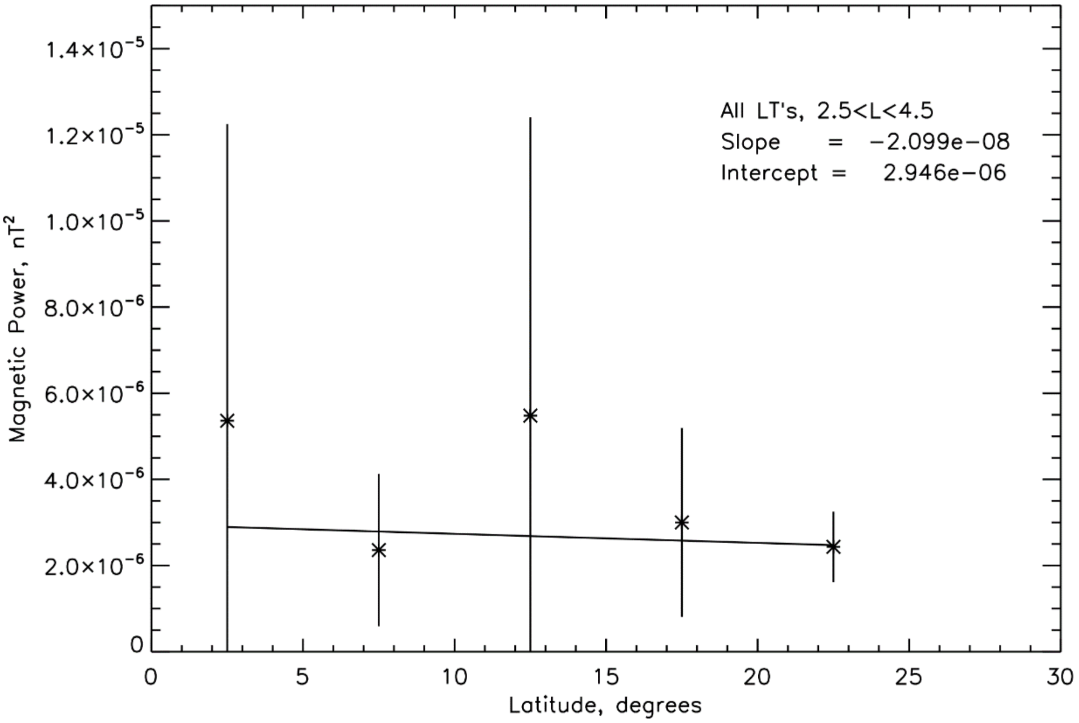

Supplementary Figure 1. Variation of chorus wave power with latitude averaged over all local times and L-shells between 2.5 and 4.5. The slope and intercept are for a weighted linear least squares fit to the data (stars indicate data means).

The variation of wave power with frequency relative to the equatorial gyrofrequency, $\beta = f/f_{ce}$, is shown in Supplemental Figure 2. The line shows the weighted least squares fit to a Gaussian curve. The parameters quoted on the figure give the power as a Gaussian fit over relative frequency

$$P_\beta = P_0 \exp\left\{-\frac{(\beta - \beta_0)^2}{\beta_w^2}\right\}$$

Where β_0 is the relative frequency where the Gaussian peaks and β_w is the width of the Gaussian.

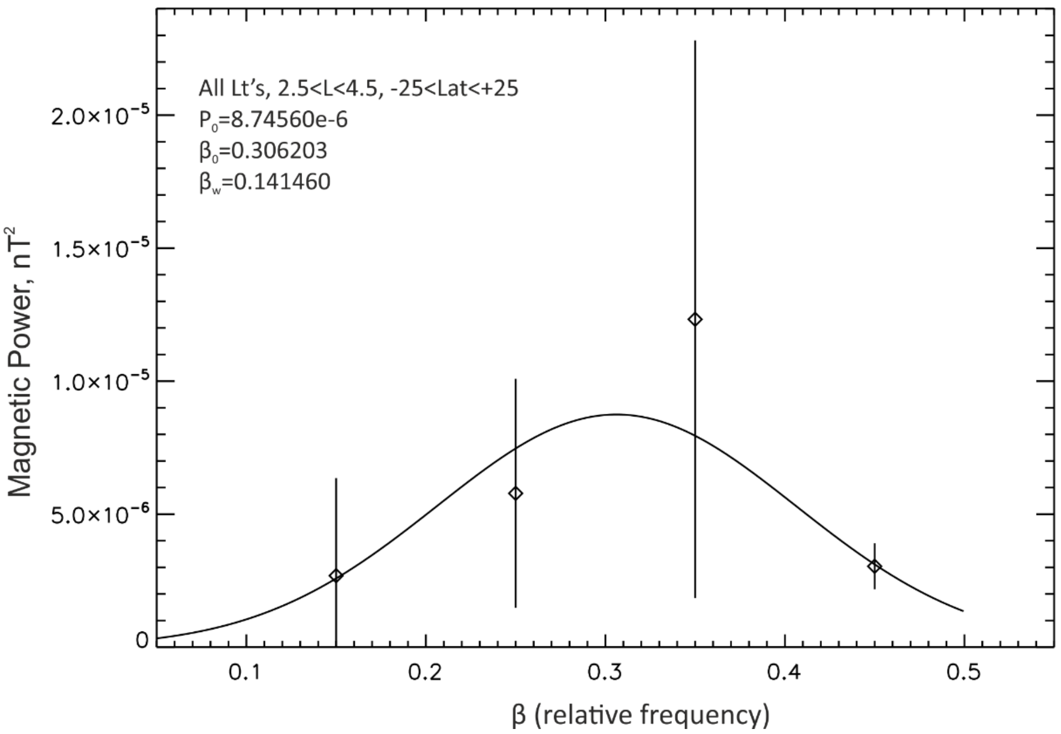

Supplementary Figure 2. Variation of chorus wave power with wave frequency normalised to the gyrofrequency averaged over all local times, latitudes up to |25°| and L-shells between 2.5 and 4.5.

Calculation of Atmospheric Scattering Diffusion Coefficient

The calculations for the pitch angle diffusion coefficient from atmospheric scattering is based on the work of Abel and Thorne [1998]. The atmospheric data on density and temperature for Saturn is based on the paper by Moore *et al.* [2009]. Species included in the calculation were H, H_2, He, H_20, CH_4, H^+, H_2^+, H_3^+, He^+, H_xO^+ (assumed to be H_20^+), CH_x^+ (assumed to be CH_4^+).

The minimum scattering angle, η, for the calculations is taken from Walt and Farley [1976] (M. Walt and T. A. Farley, Fundamentals of Cosmic Physics **2**, p1-110, 1976) and is different for ions and neutrals.

$$\eta_{ions} = \hbar/(m_r \beta c \lambda_D)$$

$$\eta_{neutrals} = \frac{2.1Z^{1/3}(1-\beta^2)^{1/2}}{137\beta}$$

where $m_r = mm_s/(m+m_s)$, m and m_s are the mass of an electron and the scattering particle respectively, c, is the speed of light, $\hbar$ is the reduced Planck constant, Z is the atomic number and β is the electron velocity/c. The Debye length, λ_D, is given by

$$\lambda_D = \sqrt{\frac{\mathcal{E}_0 kT}{n_e e^2}}$$

Where $\mathcal{E}_0$ is the permittivity of free space, k is the Boltzmann constant, n_e, is the electron density, e is the electron charge and T is the temperature of the electrons.

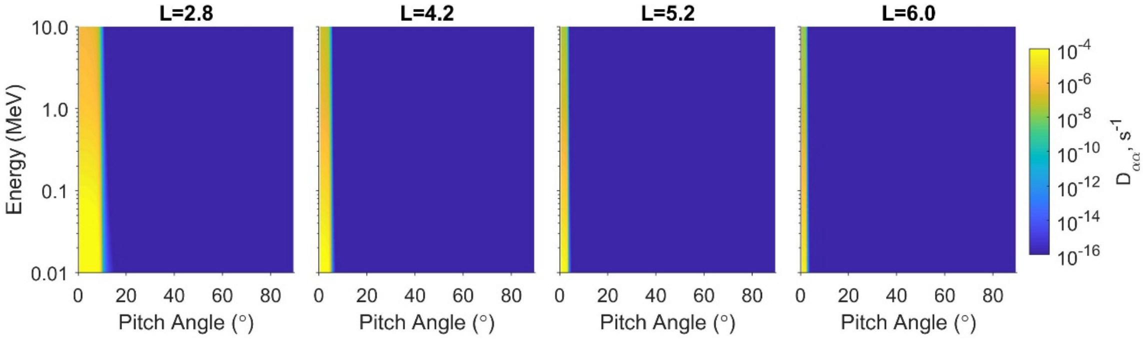

Supplementary Figure 3. Pitch angle diffusion coefficients for collisions with the atmosphere at the L-shells shown in Figure 2 in the main text. The diffusion is very low except where the pitch angle of the electron is sufficiently low that it can collide with atmospheric constituents.

PADIE and the Resonance Cone

When whistler mode chorus waves approach the resonance cone the waves change character from electromagnetic to electrostatic. The PADIE code [Glauert and Horne, 2005] uses the magnetic wave power to calculate diffusion coefficients and therefore must exclude wave particle interactions close to the resonance cone where there is no magnetic field component in the wave power.

To do this we make use of the fact that the electric field of a purely electrostatic wave is entirely parallel to the wave vector and therefore the electric field transverse to the wave vector, E_T, is zero. As the wave normal angle approaches the resonance cone the waves become increasingly electrostatic and E_T tends towards zero.

The function $g(\chi)$ in PADIE gives the variation of the wave magnetic field energy with wave normal angle, ψ, described as a Gaussian function of $\chi = \tan(\psi)$ [Glauert and Horne, 2005]. We alter this function to include the fraction of the wave power that has magnetic field energy, i.e. $(E_T/|E|)^2$ such that $g(\chi)$ becomes

$$g(\chi) = \begin{cases} \left(\frac{E_T}{|\mathrm{E}|}\right)^2 \exp\left(-\left(\frac{\chi - \chi_m}{\chi_w}\right)^2\right), & \chi_{min} \leq \chi \leq \chi_{max} \\ 0, & otherwise \end{cases}$$

Where χ_m is the value of χ at the peak of the Gaussian and χ_w is the width, χ_{min} and χ_{max} are user defined upper and lower limits.

This extra factor in $g(\chi)$ reduces the wave power as the wave normal angle approaches the resonance cone (this approach is independent of wave mode). The additional factor in $g(\chi)$ will result in an underestimate of the diffusion coefficients since the electrostatic waves have their own contribution to the diffusion. However, since the majority of the extra diffusion reported in this paper is from the $n = 0$ resonance away from the resonance cone, this underestimate is expected to be small.

Evidence for MeV Electron Butterfly Pitch Angle Distributions

There is limited data published on the pitch angle distribution of very high energy (MeV) electrons at Saturn. However, we have used the analysis of Paranicas *et al*. [2010] to produce pitch angle distributions from the Saturn Orbit Insertion data. Specifically we have plotted the data from their Figure 2 in the format of pitch angle distributions at different L-shells (see Supplementary Figure 4).

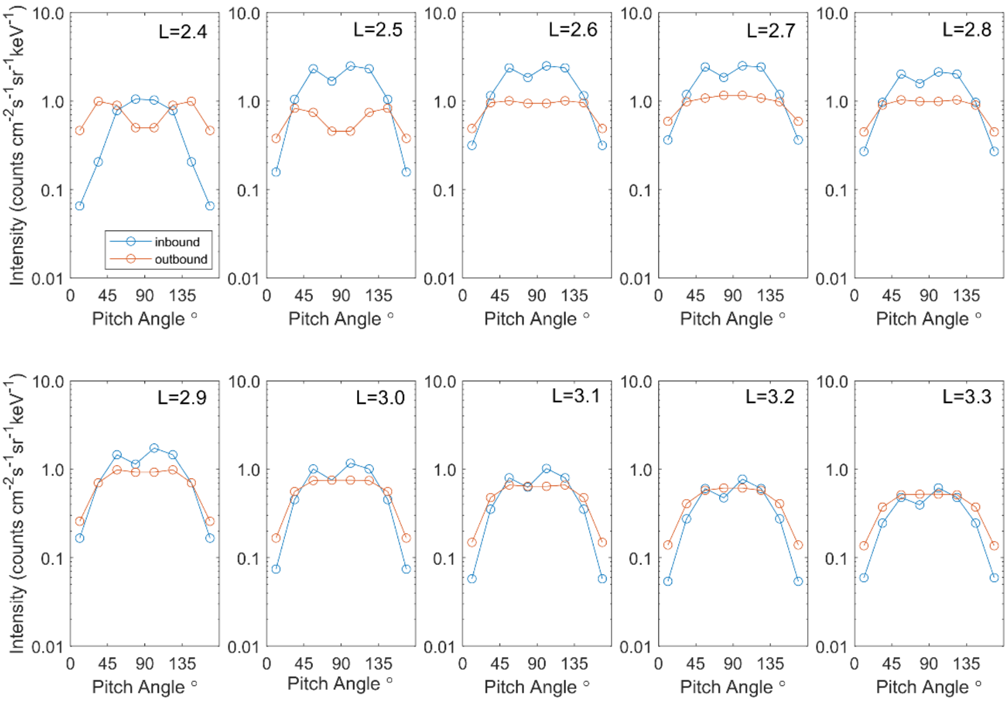

Supplementary Figure 4. Pitch angle distributions from MIMI LEMMS 1.6 to 21 MeV channel from Cassini Saturn Orbit Insertion data (using data from Figure 2 of Paranicas *et al.* [2010]). The inbound pass is shown in orange and the outbound in blue.
